# Using a Machine Learning Algorithm to Predict the Likelihood of Presence of Dental Caries among Children Aged 2 to 7

**DOI:** 10.3390/dj9120141

**Published:** 2021-12-01

**Authors:** Francisco Ramos-Gomez, Marvin Marcus, Carl A. Maida, Yan Wang, Janni J. Kinsler, Di Xiong, Steve Y. Lee, Ron D. Hays, Jie Shen, James J. Crall, Honghu Liu

**Affiliations:** 1 Section of Pediatric Dentistry, Division of Growth & Development, School of Dentistry, University of California, Los Angeles, CA 90095, USA; jnhaiem@dentistry.ucla.edu; 2 Division of Public Health & Community Dentistry, School of Dentistry, University of California, Los Angeles, CA 90095, USA; mmarcus@dentistry.ucla.edu (M.M.); cmaida@dentistry.ucla.edu (C.A.M.); dixiong@g.ucla.edu (D.X.); shenjie@stat.ucla.edu (J.S.); jcrall@dentistry.ucla.edu (J.J.C.); hhliu@dentistry.ucla.edu (H.L.); 3 Division of Oral Biology and Medicine, School of Dentistry, University of California, Los Angeles, CA 90095, USA; 4 Division of Infectious Diseases, David Geffen School of Medicine, University of California, Los Angeles, CA 90095, USA; wangyan@ucla.edu; 5 Department of Biostatistics, School of Public Health, University of California, Los Angeles, CA 90095, USA; 6 Division of Constitutive and Regenerative Sciences, School of Dentistry, University of California, Los Angeles, CA 90095, USA; slee@dentistry.ucla.edu; 7 Department of Health Policy and Management, School of Public Health, University of California, Los Angeles, CA 90095, USA; drhays@ucla.edu; 8 Division of General Internal Medicine and Health Services Research, Department of Medicine, University of California, Los Angeles, CA 90095, USA

**Keywords:** dental caries, children, oral health, disparities, machine learning algorithms, random forest

## Abstract

Background: Dental caries is the most common chronic childhood infectious disease and is a serious public health problem affecting both developing and industrialized countries, yet it is preventable in most cases. This study evaluated the potential of screening for dental caries among children using a machine learning algorithm applied to parent perceptions of their child’s oral health assessed by survey. Methods: The sample consisted of 182 parents/caregivers and their children 2–7 years of age living in Los Angeles County. Random forest (a machine learning algorithm) was used to identify survey items that were predictors of active caries and caries experience. We applied a three-fold cross-validation method. A threshold was determined by maximizing the sum of sensitivity and specificity conditional on the sensitivity of at least 70%. The importance of survey items to classifying active caries and caries experience was measured using mean decreased Gini (MDG) and mean decreased accuracy (MDA) coefficients. Results: Survey items that were strong predictors of active caries included parent’s age (MDG = 0.84; MDA = 1.97), unmet needs (MDG = 0.71; MDA = 2.06) and the child being African American (MDG = 0.38; MDA = 1.92). Survey items that were strong predictors of caries experience included parent’s age (MDG = 2.97; MDA = 4.74), child had an oral health problem in the past 12 months (MDG = 2.20; MDA = 4.04) and child had a tooth that hurt (MDG = 1.65; MDA = 3.84). Conclusion: Our findings demonstrate the potential of screening for active caries and caries experience among children using surveys answered by their parents.

## 1. Introduction

Dental caries is the most common chronic childhood infectious disease and is a serious public health problem affecting both developing and industrialized countries, yet it is preventable in most cases [1,2,3,4,5,6]. In the United States (US), the overall prevalence of dental caries has decreased in recent years, but there has been an increase among toddlers and preschoolers 5 years old and younger [4,7,8]. Early childhood caries (ECC) currently affects 21% of 2- to 5-year-old US children [9]. If left untreated, dental caries can lead to infections, pain and loss of teeth, and interfere with daily activities like eating, sleeping, learning and playing [7,10,11].

While numerous factors contribute to ECC, such as diet, oral hygiene habits and oral bacteria, it can be prevented with appropriate behavior modifications [4,6,7,8,9]. Although oral health education is not the only step in ECC prevention (community water fluoridation and health-promoting oral health policies are equally significant), it is a critical and important step [7,12]. Oral health education traditionally falls within the scope of responsibilities of dental providers and public health educators. However, educating parents on how to assess their children’s oral health status is crucial to identifying early signs of ECC, such as enamel opacity and high levels of plaque, thus helping to reduce the incidence and prevalence of ECC.

Involving parents in their children’s oral health care is more important now than ever as the current COVID-19 pandemic provides an important lesson on how a single event can impact the way in which preventive health care is delivered [13]. During this pandemic, many in-person preventive and non-urgent dental care appointments had to be canceled. While telehealth options were made available, providers had to depend more on parent’s assessment of their children’s oral health status as they could not do the actual exam themselves. Additionally, the current trend of teledentistry and virtual dental homes will rely less on in-person routine preventive dental visits/screenings to identify problems with children’s teeth and more on parents to assess the status of their children’s teeth [13]. Thus, understanding parent’s knowledge and perceptions of their child’s oral health care can provide critical information to oral health providers when in-person visits are not possible.

The authors’ previous work showed that several survey items completed by children and their parents were significantly associated with the oral health status index, including items related to perception of dental appearance and physical factors of a child’s mouth, such as being pleased/happy with the look and color of the child’s teeth, the child having straight versus crooked or crowded teeth and the child having pain in the mouth/teeth [14,15,16]. This suggests survey items have the potential to be used to assess the oral health status of children when in-person dental screenings are not possible. While these studies and others found that children 8 years and older could accurately report on their own health, infants and young children under the age of 8 must rely on their parents to assess their oral health status [15,17,18,19,20,21,22].

This study will help fill this gap by utilizing random forest (RF), a machine learning algorithm, to identify the best set of items from the parent’s oral health survey that predict the likelihood of the presence of active caries and caries experiences for children between 2 to 7 years of age and conducting a dental exam to document active caries and caries experience to identify decayed teeth, missing teeth, filled teeth and tooth (DMFT) position [23].

## 2. Materials and Methods

### 2.1. Sample

The sample for this paper included 182 parents and their child between 2 and 7 years of age. This is a subsample of a larger study that recruited about 600 adolescents and children between 2 and <18 years of age and their parents from August 2015 through October 2019 to develop a set of oral health items to predict oral health status [15]. Parents of all children between 2 and <18 years of age and children between 8 and <18 years of age completed a survey using Audio Computer Assisted Self-Interview Software (ACASI). Parents and their children were selected from 12 dental care sites throughout Los Angeles County. The sites included community dental clinics, comprehensive health centers and group and solo general and pediatric practices. To obtain a diverse sample, these sites covered different geographic areas and communities, ranging from low-income underserved immigrant neighborhoods to high-income professional communities with diverse racial and ethnic compositions. Inclusion criteria for recruitment included that only one child per family could be enrolled in the study. Children in orthodontic treatment were excluded from the study as this would interfere with a dental examination. For the current study, we evaluated survey items answered by the 182 parents of children 2 to 7 years of age (representing 30% of all participants from the larger study). For a complete description of the methodology, please refer to the authors’ previous work. [14,15]. Institutional review board approval for this study was obtained from the University of California, Los Angeles, Office of the Human Research Protection Program (Institutional Review Board approval 13-001330). Written consent was obtained from parents prior to participation. This study conforms to the Strengthening the Reporting of Observational Studies in Epidemiology (STROBE) guidelines for cross-sectional studies [24,25].

### 2.2. Data Collection

Parents completed a 34-item questionnaire using ACASI. The questionnaire included self-reported oral health-related items assessing physical health, mental health and social function domains. The questions were based on the researchers’ previous work on the multi-level influences on oral health which integrates the life-course concept into the dynamics of oral health by including genetic, biological, behavioral, social and economic contexts that change as a person develops through childhood, adolescence, young adulthood and later adult life [15,16,26]. Specific topic areas for children 2–7 years of age were use of fluoridated tap water and fluoridated toothpaste, access to dental services (including fluoride varnish applications), oral health status, physical characteristics of teeth and gums, general well-being, health preventive actions taken by parents for their children, tooth brushing habits, oral hygiene and socio-demographics [15,16,26]. Most of the respondents were parents, but in a few cases the respondent was a caregiver such as a grandparent. Our analyses did not distinguish by type of respondent. The questionnaire was only available in English, but Spanish and Chinese translators were available on-site for assistance, if needed. It took parents an average of 20–45 min to complete the questionnaire. Parents were compensated with USD 55 in cash if they completed the questionnaire and the child was screened by a dentist.

Children received clinical examinations prior to or after parents completed the questionnaire. The clinical examination included a full mouth examination of all primary and permanent teeth. It consisted of examining the oral mucosa, teeth for the presence of obvious caries and decalcification (white spots), the presence of plaque on the centrals and molars, when present, and bleeding on probing and inflammation of the gingiva. The dental examiners were two faculty dentists from the UCLA School of Dentistry who underwent training and inter- and intra-rater calibration, which was analyzed using Cohen’s kappa. The intra-rater calibration was conducted by duplicate dental examinations on the same child between two dentists. We assessed the intra- and inter-examiners during the training stage at each of the study sites. A total of 51 children received dental exams by both examiners to check the inter-rater reliability. In each study site, 3 children were randomly selected to be checked by the two pediatric dentists, but the same recorder. All the clinical exam measurements were repeated. For active caries (coded as DT), 2 subjects were recorded as having DT (DT > 0) by both examiners and 44 subjects were recorded as having no caries (DT = 0). The percentage agreement that both the examiners identified a child with active caries or not was 88%. However, with an imbalanced distribution of the presence of active caries in our study (13%), the high agreement did not align with the low kappa (0.39), which is common with an imbalanced distribution of an outcome [27]. In these cases, using percentage agreement between two examiners is appropriate [27]. For caries experience, a total of 23 children with caries experience (DMFT > 0) and another 23 children without caries experience were found by both dental examiners. The percentage agreement between the two examiners was 90% with a kappa of 0.80. The results of the dental exams were based on the presence of active caries (DT coded as Yes/No) and the DMFT index which has been evaluated in previous studies with children for caries experience [23]. The DMFT/dmft index was coded as 0 for no caries experience and 1 for one or more teeth with decay, missing or with fillings. This includes both primary and permanent teeth. We did not include white spots, crookedness, fracture or abnormal teeth positions.

### 2.3. Data Analysis

We used RF to derive the machine learning algorithm, which is based on a collection of decision trees [28,29]. The decision trees are obtained by searching for variables within the training set and splitting them in such a way that will generate the “best” two subsets [28,29]. The goal is to create branches and leaves based on an optimal splitting criterion. Specifically, at every branch or node, a conditional statement classifies the data point based on a fixed threshold in a specific variable, therefore splitting the data. To make predictions, every new instance starts in the root node (top of the tree) and moves along the branches until it reaches a leaf node where no further branching is possible. In prediction, more than one tree is needed to improve the accuracy [28,29]. RF is based on the summary statistics of all prediction trees. It is capable of fitting complex datasets and performing both classification and regression tasks [28].

In developing the RF, 70% of subjects were assigned to a training set and 30% of subjects to a testing set randomly based on the outcome variables (active caries and caries experience) [28,29]. Due to the small sample size and low prevalence of active caries, the models were developed on the training sets using a 3-fold cross-validation method. Cross-validation is a technique used to help tune the parameters using the training set only [28,29,30]. We divided the training set into 3 folds randomly and equally. For each cross-validation loop, 2 out of 3 folds were selected as the subtraining set and the remaining fold as the validation set [28,29,30]. The model is derived from 2 out of the 3 folds and validated on the withholding validation set. It was repeated 3 times so that each fold could be treated as the validation set only once. The results of the 3 validation sets were aggregated together as the training model [28,29,30]. We derived parameters for each decision tree (mtry), and number of decision trees to grow (ntree) on the best cross-validation aggregated area under the receiver operating characteristic curve (AUC) to obtain the best models. A threshold is determined by maximizing the sum of sensitivity (true positive rate) and specificity (true negative rate) with the condition that sensitivity is at least 70%. The classification performance is evaluated on the testing set [28,29,30]. Table 1 presents the parameters and performance on cross-validation sets and testing sets separately for active caries and caries experience models.

The importance of the questionnaire items in classifying the different oral health status was measured by the mean decreased accuracy (MDA) and the mean decreased Gini coefficient (MDG) [29,30]. The MDA refers to the reduced accuracy in classification by excluding the corresponding questionnaire item. MDA is calculated on the dataset by randomly shuffling the data for that particular item and then subtracting the two accuracies (i.e., before and after shuffling). The MDG was defined by the consistency of classification by the corresponding questionnaire item. MDG decrease is calculated based on the mean decrease in Gini (i.e., *pi*(*i* − *pi*)) each time when the tree is split on that item. The value is sometimes higher because the r package weights the impurities by the raw counts, not the proportions. Both MDA and MDG are unitless. For MDA, it is measured as the ratio of correct classification to total records. For MDG, it is the measurement of the cleanliness of the split. There is no cutoff range for determining MDA and MDG values [29,30]. We selected variables with higher MDA and MDG indicating the importance of the variables to our outcomes (active caries and caries experience). The selection of questionnaire items based on both MDA and MDG led to the classification that was most accurate and robust. R statistics was used for RF analyses.

**Figure 1:**
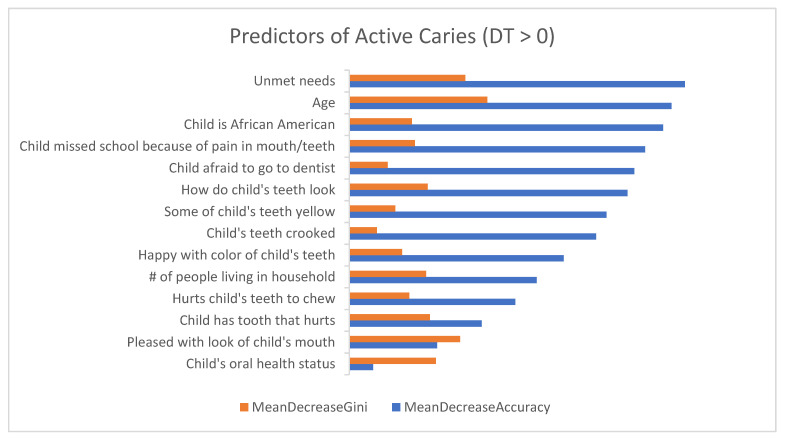
Demographic and oral health-related predictors of active caries (DT > 0) showing both the mean decreasing of Gini (MDG) and mean decreasing of accuracy (MDA) measures. The higher the MDG and MDA, the more important the variables.

**Figure 2:**
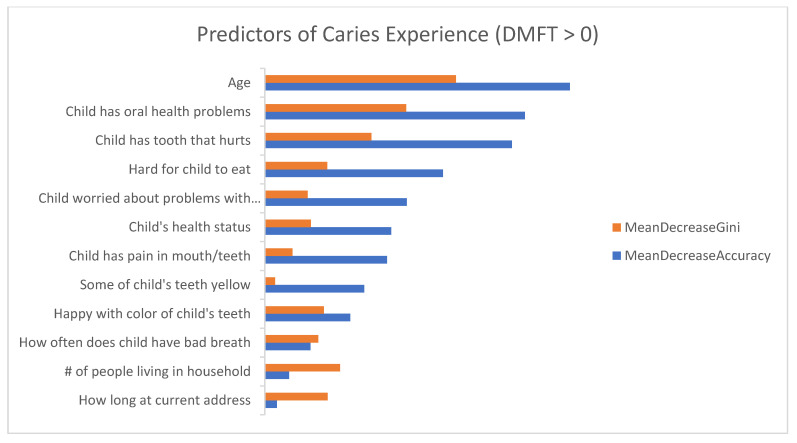
Demographic and oral health-related predictors of caries experience (DMFT > 0) showing both the mean decreasing of Gini (MDG) and mean decreasing of accuracy (MDA) measures. The higher the MDG and MDA, the more important the variables.

Figure 1 and Figure 2 show the list of questionnaire items on the *y*-axis and the importance on the *x*-axis. The longer bar indicates the more important the question is to the outcome variable.

## 3. Results

### 3.1. Sample Characteristics

The descriptive statistics for the demographics, outcome variables and oral health-related predictor variables are presented in Table 2. The majority of parents/caregivers were female (*n* = 126; 69%), 33 to 44 years of age (*n* = 115; 63%) and Hispanic/Latino (*n* = 71; 39%), Caucasian/White (*n* = 45; 24%), Asian (*n* = 21; 11%) and African American (*n* = 14; 8%). Approximately half the children were between the ages of 2 and 4 (*n* = 76; 42%). Parents identified their child’s race/ethnicity as: Hispanic/Latino (*n* = 71; 39%), Caucasian/White (*n* = 43; 24%), Asian (*n* = 21; 12%), African American (*n* = 14; 7%) and multi-racial (*n* = 21; 12%).

Based on the clinical dental exam, 13% (*n* = 23) of children had active caries and 47% (*n* = 86) had more than one caries experience based on the DMFT index.

### 3.2. Questionnaire Items Predicting Active Caries

Figure 1 presents the list of important questionnaire items for classifying children with active caries. The top 10 questionnaire items from the RF model that were the highest predictors of active caries (had the highest MDG and MDA) included: Parent’s age (MDG = 0.84; MDA = 1.97), unmet needs (MDG = 0.71; MDA = 2.06), parents were pleased or happy with the look of their child’s mouth/teeth/jaws/gums (MDG = 0.68; MDA = 0.54), child’s overall oral health status (MDG = 0.53; MDA = 0.14), child has a tooth that hurts (MDG = 0.50; MDA = 0.81), overall look of child’s teeth (MDG = 0.48; MDA = 1.70), number of people living in the household (MDG = 0.47; MDA = 1.15), child missed school because of pain in mouth/teeth (MDG = 0.40; MDA = 1.81), child is African American (MDG = 0.38; MDA = 1.92) and child’s teeth hurt when they chew (MDG = 0.37; MDA = 1.02).

### 3.3. Questionnaire Items Predicting Caries Experience (Based on DMFT Index)

Figure 2 presents the list of important questionnaire items for classifying children with caries experience. The top 10 questionnaire items from the RF model that were the highest predictors of caries experience included: Parent’s age (MDG = 2.97; MDA = 4.74), child had an oral health problem within past 12 months (MDG = 2.20; MDA = 4.04), child had tooth that hurts (MDG = 1.65; MDA = 3.84), number of people living in household (MDG = 1.17; MDA = 0.38), how long parent and child have lived at current address (MDG = 0.98; MDA = 0.19), hard for child to eat because of pain in his/her mouth (MDG = 0.97; MDA = 2.77), parent is happy with the color of their child’s teeth (MDG = 0.92; MDA = 1.33), how often child has bad breath (MDG = 0.83; MDA = 0.71), child’s overall health status (MDG = 0.72; MDA = 1.97) and child worried about problems with teeth/mouth (MDG = 0.66; MDA = 2.21).

The accuracy, sensitivity and specificity for the RF algorithm’s 3-fold cross-validation set for active caries and caries experience are 0.71, 0.94 and 0.68 for active caries and 0.71, 0.78 and 0.64 for caries experience (Table 1).

## 4. Discussion

In this study, we used RF to identify questionnaire items that were predictors of active caries and caries experiences using the DMFT index among 2 to 7-year-old children.

This is the most recent of multiple papers that develop oral health algorithms or tools which could be used by dentists, oral health researchers and professionals and public policy makers for oral health screening, program assessment, oral health evaluation and oral health management and policy planning [14,15,16,17,18,26]. The studies in this series on children between 8 and 17 years of age (where both parents and children completed the questionnaire) [14,15,16,17,18] showed similar predictors of oral health as we found in our study among children between 2 and 7 years of age (where only the parents completed the questionnaire). For example, the current study showed that dental appearance and aesthetics, such as being pleased/happy with the look of the child’s teeth/mouth/gums and color of the child’s teeth (tooth color, such as white, yellow or brown, is associated with oral hygiene and is considered a reflection on a person’s ability to engage in self-care) [17] were important predictors of dental caries among children between 2 and 7 years of age, similar to what was found in the study among children between 8 and 17 years of age [14,15,16,17,18]. Physical aspects of a child’s mouth, such as the child having a tooth that hurts and the child having difficulty eating due to pain in the mouth/teeth were also important predictors of the presence of caries and overall oral health among children between 2 and 7 years of age, similar to what was found in the study among children between 8 and 17 years of age [14,15,16,17,18]. These findings indicate that oral health questionnaire items could be used by pediatric dentists and other oral health care professionals to predict the presence of caries and other oral health-related problems in children. Identifying key predictors of dental caries from machine learning algorithms also gives the clinician an opportunity to educate patients/caregivers on the importance of good oral hygiene behaviors and a healthy diet to preventing childhood caries since many of the questionnaire items focus on the prevention of childhood caries [31,32].

This study also showed that unmet needs and demographic characteristics such as parent’s age, child’s race (African American), number of people living in the household and how long the parent and child have lived at their current address were strong predictors of active caries and caries experience in children between 2 and 7 years of age. These findings indicate that socio-demographic factors are important predictors of active caries and caries experience, which is not surprising as factors such as race/ethnicity and family income (which can affect household structure) are known social determinants of health that are associated with oral health disparities resulting in dental caries and untreated dental caries [2,5,6,8]. Having this information will give dental professionals the opportunity to conduct a more in-depth caries risk assessment of their patients’ oral health-related needs, which traditionally includes a combination of questions pertaining to caries-related biological, social and cultural predisposing risk factors, disease indicators and protective factors, and make appropriate referrals to social-related services, if needed [32].

The study had many limitations, mainly the small sample size (*n* = 182) and low percentage of children with active caries (13%) which limited the identification of variables that were predictors of active caries. To adjust for this, we used the RF methodology which works well for non-linear and high-dimensional variable sets and has a good sensitivity and relative acceptable specificity. This study required parents/caregivers to complete a questionnaire about their young child’s oral health which introduces different types of bias, such as social desirability bias and potential response bias if the respondent was a grandparent rather than a parent. While patient reported outcomes directly from children are the gold standard for questionnaire items, patient proxy reported outcomes by the parent/caregiver are useful when children are too young and do not yet have the cognitive ability to self-report [15,22,33]. Selection bias is also possible given that parents/caregivers were given monetary incentives to participate. Finally, this study was conducted with families who were recruited from dental clinics and practices that agreed to participate in the study and may not be generalizable to families who are not currently under dental care.

## 5. Conclusions

Our findings demonstrate how the use of machine learning algorithms based on oral health surveys can help dental providers identify key predictors of dental caries in infants and young children. Once the key predictors of dental caries have been identified, dental providers can then include these items as part of their caries risk assessment and take the opportunity to educate their patients/caregivers on the importance of good oral hygiene behaviors. Additionally, the emerging trend of teledentistry and virtual homes will rely less on in-person routine preventive dental visits and more on having parents assess the current status of their children’s teeth and oral health by asking them some key questions that are known to have high predictability for outcomes of interest such as active caries and caries experience. Thus, the development of algorithm “toolkits” that help dental professionals assess their patient’s oral health could prove extremely useful for prevention of dental caries among children.

## Figures and Tables

**Table 1:** Random forest performance on cross-validation sets and testing sets for active caries and caries experience.

			3-Fold Cross-Validation			Test		
	Tuning Parameter *	Threshold	Accuracy	Sensitivity	Specificity	Accuracy	Sensitivity	Specificity
Active Caries	mtry = 41; ntree = 100	0.08	0.71	0.94	0.68	0.62	0.57	0.63
Caries Experience	mtry = 2; ntree = 100	0.36	0.71	0.78	0.64	0.73	0.92	0.55

* 1. mtry stands for the number of variables for each tree; 2. ntree stands for the number of total trees to grow; 3. threshold is used to classify the instances into two groups.

**Table 2:** Descriptive characteristics of main outcomes, demographics and oral health-related predictor variables (*n* = 182).

Sample Characteristics	*n* (*%*)
Main Outcome Variables	
Active Caries	
Yes	23 (13)
No	159 (87)
Caries Experience (DMFT)	
Yes	86 (47)
No	96 (53)
Parent Demographics	
Age, years	
<30	42 (23)
30–44	115 (63)
≥45	25 (14)
Gender	
Male	56 (31)
Female	126 (69)
Race/ethnicity	
Caucasian/White	45 (24)
African American	14 (8)
Hispanic/Latino	71 (39)
Asian	21 (11)
Other	31 (18)
Number of people in household	
≤3	31 (17)
4–5	101 (56)
≥6	50 (27)
Number of years lived at current address	
≤1 year	33 (18)
>1 year–≤5 years	82 (45)
>5 years–≤10 years	36 (20)
>10 years	31 (17)
Child’s Demographics	
Age, years	
2	21 (12)
3	25 (14)
4	30 (16)
5	22 (12)
6	44 (24)
7	40 (22)
Gender	
Male	93 (51)
Female	89 (49)
Race/ethnicity	
Caucasian/White	43 (24)
African American	14 (7)
Hispanic/Latino	71 (39)
Asian	21 (12)
Multi-racial	21 (12)
Other	12 (6)
Oral health-related predictor variables (survey questions)	
In general, would you say your child’s oral health status is:	
Excellent/very good	120 (66)
Good	50 (27)
Fair/poor	12 (7)
During the last 12 months, did your child have an oral health problem?	
Yes	55 (30)
No	127 (70)
In the last 4 weeks, how much of the time were you pleased or happy with the look of your child’s mouth, teeth, jaws or gums?	
Always/almost always	149 (82)
Often/sometimes	30 (16)
Almost never/never	3 (2)
In the last 4 weeks, how much of the time did your child have pain or discomfort with his/her mouth, tongue, teeth, jaws or gums?	
Always/almost always	0 (0)
Often/sometimes	14 (8)
Almost never/never	168 (92)
How often does your child have bad breath?	
Always/almost always	10 (6)
Often/sometimes	91 (50)
Almost never/never	81 (44)
When I look at my child’s teeth	
They look fine	119 (66)
They could look a little better	48 (26)
They could look a lot better	15 (8)
In the last 4 weeks, how much of the time was your child worried or concerned about problems with his/her mouth, tongue, teeth, jaws or gums?	
Always/almost always	1 (1)
Often/sometimes	7 (4)
Almost never/never	174 (95)
My child’s mouth hurts	
Always/almost always	0 (0)
Often/sometimes	9 (5)
Almost never/never	173 (95)
My child has a tooth that hurts	
Always/almost always	1 (1)
Often/sometimes	15 (8)
Almost never/never	166 (91)
It hurts my child’s teeth to chew	
Always/almost always	0 (0)
Often/sometimes	7 (4)
Almost never/never	175 (96)
It is hard for my child to eat because of pain in his/her mouth	
Always/almost always	0 (0)
Often/sometimes	9 (5)
Almost never/never	173 (95)
How happy are you with the color of your child’s teeth?	
Very much/quite a bit	141 (78)
Somewhat	24 (13)
A little bit/not at all	17 (9)
Some of my child’s teeth are yellow	
Yes	21 (12)
No	161 (88)
My child’s teeth are crooked	
Yes	15 (8)
No	167 (92)
During the past 12 months, was there a time that your child needed dental care, but did not get it?	
Yes	8 (4)
No	174 (96)
How much is your child afraid to go to a dentist?	
Not at all	97 (53)
A little bit/somewhat	76 (42)
A great deal	9 (5)
During the last school year, how many days of school did your child miss because of pain in his/her mouth, teeth, gums (if child goes to school)?	
Never	168 (92)
1 to 3 days	13 (7)
4 days or more	1 (1)

## Data Availability

The data presented in this study are available on request from the corresponding author [F.R.-G.] or Principal Investigator [H.L.]. The data are not publicly available due to privacy and ethical issue.
